# Mental health and lifestyle habits among healthcare workers

**DOI:** 10.1192/j.eurpsy.2025.1874

**Published:** 2025-08-26

**Authors:** I. Sellami, A. Abbes, A. Feki, A. Haddar, H. Halweni, M. Hajjaji, S. Baklouti, M. L. Masmoudi, K. Jmal Hammami

**Affiliations:** 1 Occupationnal medecine; 2 Rheumatology, CHU Hedi Chaker; 3 General practice, Medecine university, Sfax, Tunisia

## Abstract

**Introduction:**

In the health field, healthcare workers (HCWs) need to be fulfilled to improve the quality of care. The mental health of these workers can affect the practice of their lifestyle habits.

**Objectives:**

Our study aims to assess the relationship between mental health and lifestyle habits among HCWs.

**Methods:**

We conducted a descriptive, analytical and cross-sectional survey among HCWs using a self-administrated questionnaire. We collected socio-professional data. We assessed mental health using the depression anxiety and stress scale (DASS 21).

**Results:**

Our study included 200 healthcare workers, 71% of whom were female. The average age of participants was 42.9 years, with an average job tenure of 14.2 ± 10.1 years. We found that 12.5% of participants were smokers, 5% were former smokers, and 20.5% were passive smokers. Three participants were alcoholics, and none used drugs or chewed Neffa. Additionally, 32% of the population engaged in sports, with an average duration of 4.5 ± 2.9 hours per week.

According to the DASS21, 63% of participants exhibited symptoms suggestive of anxiety, 65.5% showed signs of stress, and 39.5% had depressive symptoms. We found that participation in sporting activities was associated with reduced anxiety (p = 0.04).

**Conclusions:**

Our findings highlight a correlation between reduced anxiety and practicing sporting activities. It is crucial to encourage HCWs to maintain regular physical activity to promote an active lifestyle, reduce stress and improve mood in order to enhance the quality of care.

**Disclosure of Interest:**

None Declared

